# hsa-miR-181a-5p inhibits glioblastoma development via the MAPK pathway: *in-silico* and *in-vitro* study

**DOI:** 10.32604/or.2024.051569

**Published:** 2024-11-13

**Authors:** MAHDI ABDOLI SHADBAD, BEHZAD BARADARAN

**Affiliations:** 1 Student Research Committee, Tabriz University of Medical Sciences, Tabriz, Iran; 2 Immunology Research Center, Tabriz University of Medical Sciences, Tabriz, Iran

**Keywords:** Glioblastoma, hsa-miR-181a-5p, MAPK, MicroRNAs

## Abstract

**Background:**

Glioblastoma remains a highly invasive primary brain malignancy with an undesirable prognosis. Growing evidence has shed light on the importance of microRNAs (miRs), as small non-coding RNAs, in tumor development and progression. The present study leverages the *in-silico* and *in-vitro* techniques to investigate the significance of hsa-miR-181a-5p and the underlying hsa-miR-181a-5p-meidated signaling pathway in glioblastoma development.

**Methods:**

Bioinformatic studies were performed on GSE158284, GSE108474 (REMBRANDT study), TCGA-GTEx, CCLE, GeneMANIA, Reactome, WikiPathways, KEGG, miRDB, and microT-CDS to identify the significance of hsa-miR-181a-5p and its underlying target. Afterward, the U373 cell line was selected and transfected with hsa-miR-181a-5p mimics, and the cell viability, clonogenicity, migration, mRNA expression, apoptosis, and cell cycle were studied using the MTT assay, colony formation test, migration assay, qRT-PCR, and flow cytometry respectively.

**Results:**

hsa-miR-181a-5p expression is decreased in glioblastoma samples. The *in-silico* results have shown that hsa-miR-181a-5p could regulate the MAPK pathway by targeting *AKT3*. The experimental assays have shown that hsa-miR-181a-5p decreases the migration of glioblastoma cells, arrests the cell cycle, and increases the apoptosis rate. Besides downregulating *MMP9* and upregulating *BAX*, hsa-miR-181a-5p downregulates *MET*, *MAP2K1*, *MAPK1*, *MAPK3*, and *AKT3* expression in U373 cells. The *in-vitro* results were consistent with *in-silico* results regarding the regulatory effect of hsa-miR-181a-5p on the MAPK pathway, leading to tumor suppression in glioblastoma.

**Conclusions:**

hsa-miR-181a-5p inhibits glioblastoma development partially by regulating the signaling factors of the MAPK pathway.

## Introduction

Glioblastoma is considered a brain malignancy with a poor prognosis. It has been reported that glioblastoma accounts for more than 61% of diagnosed glioma in the United States between 2000 and 2014 [1]. Glioblastoma is a highly aggressive malignancy in which tumor relapse is almost inevitable, and there is no standard clinical treatment for recurrent glioblastoma [2]. Therefore, developing novel treatments for glioblastoma is an imperative matter; a better understanding of glioblastoma biology is essential to developing novel treatments for glioblastoma patients.

MicroRNAs (miRs) are single-stranded endogenous RNAs with a length of 22 nucleotides that post-transcriptionally regulate gene expression [3]. It is estimated that miRs can regulate the expression of 90% of protein-coding genes, and one miR can regulate hundreds of genes’ expression directly and indirectly [4]. miRs combine with Argonaute and produce the miR-induced silencing complex that complementarily binds to the 3′ untranslated regions of target mRNA; this leads to mRNA degradation [5]. Indeed, miRs have significant roles in regulating various signaling pathways [6]. miR-181a-5p has tumor-suppressive properties that are dysregulated in malignancies like non-small cell lung cancer, oral squamous cell carcinoma, retinoblastoma, and esophageal squamous cell carcinoma [7–10]. Besides, hsa-miR-181a-5p expression is decreased in glioma tissues compared to non-tumoral brain tissues [11]. The comparable results regarding the downregulation of miR-181a in glioma have been reported by Ciafre et al. [12]. The dysregulated expression level of hsa-miR-181a-5p has been associated with the clinical picture of patients with glioma as well; miR-181a-5p is downregulated in recurrent glioblastoma tissues compared to primary ones [13]. Besides, it has been demonstrated that there is a negative correlation between glioma grade with hsa-miR-181a-5p expression [14]. High expression of circulating miR-181a has been linked with improved overall survival of patients with high-grade gliomas undergoing total and partial resection [15]. Huang et al. have shown that miR-181a is downregulated in glioblastoma tissues, and low expression of miR-181a is associated with poor overall survival of glioblastoma patients. Besides, increased expression of miR-181a-5p stimulates the apoptosis of glioblastoma cells [16]. Chen et al. have shown that miR-181a enhances the radiosensivity of glioblastoma cells [17]. Also, Marisetty et al. have shown that the delivery of miR-181a-5p increases the survival of immunocompetent glioma models [18]. There have been reports that miR-181a-5p overexpression inhibits the Wnt/β-catenin signaling pathway in colorectal cancer, TGF-β1/Smad pathway in hepatocellular carcinoma, and MAPK pathway in cutaneous squamous cell carcinoma [19–21]. Although the studies have reported anti-tumoral properties of hsa-miR-181a-5p in glioblastoma, its underlying mechanism is not well-established. Thus, there is a need to investigate the hsa-miR-181a-5p-mediated signaling pathway on glioblastoma development.

The advances in computational biology and RNA sequencing have brought ample opportunities for discovering dysregulated miRs in various human conditions development. Tumor bulk RNA studies between tumoral and non-tumoral tissues provide unprecedented data on the dysregulation patterns of mRNA and miRs, and bioinformatic prediction tools can predict and rank the interaction between miRs and mRNAs based on their sequences [22]. In this regard, the application of bioinformatic tools along with experimental studies can further facilitate the development of novel anti-neoplastic treatments [23]. In the current study, we leveraged bioinformatics to study the significance of hsa-miR-181a-5p in glioblastoma development. Also, the subsequent experimental analyses were performed to confirm the obtained *in-silico* results.

## Materials and Methods

In this study, we employed *in-silico* and *in-vitro* approaches to study the significance of hsa-miR-181a-5p in glioblastoma development. Regarding the bioinformatics approaches, the GSE158284, GSE108474 (REMBRANDT study), TCGA-GTEx, CCLE, GeneMANIA, Reactome, WikiPathways, KEGG, miRDB, and microT-CDS were leveraged to investigate the role of hsa-miR-181a-5p and its mediated signaling pathway in glioblastoma development. Also, the *in-vitro* assays, i.e., 3-(4,5-dimethylthiazol-2-yl)-2,5-diphenyl-2H-tetrazolium bromide (MTT), annexin V/propidium iodide (PI), scratch, colony formation, qRT-PCR, and cell cycle assays, were performed to validate the *in-silico* results. This study was approved by the Ethics Committee of Tabriz University of Medical Sciences (IR.TBZMED.VCR.REC.1402.082).

### Tumor bulk RNA analysis

In the first step, we used system biology approaches to study miR-181a expression in glioblastoma and non-cancerous brain tissues. After searching the Gene Expression Omnibus (GEO) database, the GSE158284 and GSE108474 datasets were selected for this aim. The GSE158284 dataset used Agilent-021827 Human miRNA Microarray (V3) (Probe Name version) for sequencing the miR expression of 20 primary adult glioblastoma, 9 primary pediatric glioblastoma, and 11 non-tumoral brain tissues. The GSE108474 dataset used GPL570 for sequencing mRNA in 550 brain cancers and normal brain tissues. After logarithmically transforming the raw data and the quantile normalization of the expression values, the box plots of included samples were drawn using R software (version 4.3.2). Then, the clustering analyses and heatmaps were provided to examine the expression data of tissues. Finally, the provided annotation file was used to assign probes to miRs and mRNAs. The TCGA-GTEx dataset was retrieved with the use of GEPIA to perform a comparative study of *AKT3* expression in glioblastoma and normal brain tissues. Furthermore, the CCLE dataset was used to study miR-181a expression in A172 and U87MG cell lines.

### Identifying hsa-miR-181a-5p targets

To investigate the potential effect of hsa-miR-181a-5p ectopic expression on signaling pathways, we used the miRPathDB v2.0 database (https://mpd.bioinf.uni-sb.de/overview.html) to access the Reactome, WikiPathways, and KEGG data. In this database, we considered *p*-values less than 0.01 as significant, and we only included the findings based on “strong experimental” evidence. For external validation, the miRDB and microT-CDS databases (miRDB access point: https://mirdb.org/; microT-CDS access point: https://mircarta.cs.uni-saarland.de/targets_search/) were used to obtain the potential targets of hsa-miR-181a-5p with a target score of above 89.

### Cell culture

The U373 cell line was bought from the Pasture Institute’s cell bank in Tehran, Iran. The cells were grown in RPMI-1640 supplemented with 10% fetal bovine serum (FBS) (GIBCO, Carlsbad, CA) and penicillin (100 IU/mL) streptomycin (100 µg/mL) (Sigma-Aldrich, St. Louis, MO, USA); they were then incubated at 37°C in a 95% humidified incubator with 5% CO_2_.

### hsa-miR-181a-5p mimic transfection

U373 cells were subcultured when the confluency reached 80%. The number of cells was counted in the log phase of cell growth. The cells were suspended in a cool electroporation buffer for this purpose. A Gene Pulser Electroporation device and a 0.4 cm^3^ Gene Pulser Cuvette (Bio-Rad, CA, USA) were utilized to transfect 20 pmol of hsa-miR-181a-5p (AACAUUCAACGCUGUCGGUGAGU) into 1 × 10^6^ suspended cells.

### Assessing gene expression by qRT-PCR

Consistent with our previous studies, total RNA was isolated from the cells after 48 h of transfection using the RiboEX reagent (GeneAll Biotechnology, Seoul, South Korea) following the manufacturer’s instructions [24,25]. The purity and concentration of the collected RNA were then evaluated using a NanoDrop spectrophotometer (Thermo Fisher Scientific, Lenexa, KS, USA). The cDNA was synthesized from the RNA, utilizing an AddScript cDNA Synthesis Kit and a thermal cycler system (ADDBIO, South Korea). Then, the STEP ONE PLUS qRTPCR equipment (Applied Biosystems, Foster City, USA) was used to examine the mRNA expression of *MAP2K1*, *MET*, *AKT3*, *PIK3CA*, *MAPK1*, *MAPK3*, *MMP9*, *CASP9*, and *BAX*, and the 2^−ΔΔCt^ technique was used for subsequent statistical analyses. The 18s gene was utilized as the housekeeping gene. Using the NCBI website, all pair primer sequences were examined (Table 1).

**Table 1:** The primer sequences of studied genes

Gene	Forward primer	Reverse primer	Product length	Species
MAP2K1	CAATGGCGGTGTGGTGTTC	GATTGCGGGTTTGATCTCCAG	91	*Homo sapiens*
MET	GGACATCAGAGGGTCGCTTCA	TCTGGAGACACTGGATGGGAGT	100	*Homo sapiens*
AKT3	AGAACGACCAAAGCCAAACACA	AGTCTGTCTGCTACAGCCTGG	135	*Homo sapiens*
PIK3CA	GAAGCACCTGAATAGGCAAGTCG	GAGCATCCATGAAATCTGGTCGC	146	*Homo sapiens*
MAPK1	TCTGGAGCAGTATTACGACCC	CTGGCTGGAATCTAGCAGTCT	134	*Homo sapiens*
MAPK3	CGCTTCCGCCATGAGAATGT	GGTCAGTCTCCATCAGGTCC	106	*Homo sapiens*
MMP9	ACGACGTCTTCCAGTACCGA	TTGGTCCACCTGGTTCAACT	95	*Homo sapiens*
CASP9	CTGTCTACGGCACAGATGGAT	GGGACTCGTCTTCAGGGGAA	177	*Homo sapiens*
BAX	ACCGCCTCACTCACCATCT	GACCACTCTTCCCCACACC	166	*Homo sapiens*
18s	ACCCGTTGAACCCCATTCGTGA	GCCTCACTAAACCATCCAATCGG	159	*Homo sapiens*

### MTT assay

The effect of our transfection procedure and hsa-miR-181a-5p mimics on U373 cell viability was evaluated using the MTT test. Additionally, the MTT test was used to calculate the IC50 of temozolomide. In a nutshell, a 96-well plate was seeded with 1.2 × 10^4^ U373 cells. After the first incubation time, each well received 50 μL of MTT (2 mg/mL), and the cells were incubated for an additional three hours (Sigma-Aldrich, M5655). In each well, DMSO was administered after the medium had been removed, and the plate was then placed in the incubator for 30 min (Sigma-Aldrich, D4540). Using an ELISA reader, each well’s optical density was evaluated at 570 nm after shaking for 10 min (Sunrise RC, Tecan, Switzerland).

### Annexin V/PI assay

Two groups of U373 cells were subjected to the annexin V/PI test to measure their apoptosis rates. In a six-well plate, 2 × 10^5^ transfected and untransfected U373 cells were planted in each well. The cells were stained with annexin V and PI following the manufacturer’s instructions (EXBIO, Vestec, Czech Republic) after an additional 24 h of incubation. After that, each group’s apoptosis rates were evaluated utilizing flow cytometry(MiltenyBiotecTM FACS Quant 10; MiltenyBiotec, Germany). FlowJo software (Tree Star, San Carlos, CA, USA) was used to evaluate the collected data.

### Cell cycle assay

This assay’s seeding procedure was like the annexin V/PI test. In a nutshell, the cells were trypsinized to produce single cells following the second 24-h incubation period. Following fixation with 80% ethanol, the cells were placed at −20°C overnight. The flow cytometry was utilized to assess the distribution of cells throughout the phases of the cell cycle after treatment with RNase and labeling with DAPI. The collected data were then analyzed using the FlowJo software (Tree Star, San Carlos, CA, USA).

### Migration assay

3 × 10^5^ U373 cells were seeded in each well of a 24-well plate. Using a sterile 100 μL pipette tip, the cellular monolayer was scraped after the second 24 h of incubation. Then, the cells were cultured for 0, 24, and 48 h while an inverted microscope was utilized to capture the corresponding images (Optika, XDS-3, Italy).

### Colony formation assay

In a six-well plate, 5 × 10^3^ transfected and non-transfected cells were seeded. After a 12-day incubation period, the cells were treated with paraformaldehyde and stained with crystal violet. Using an inverted microscope, the colonies were captured on camera (Optika, XDS-3, Italy).

### Statistical analysis

The analysis of the *in-vitro* data was conducted using GraphPad Prism software version 8.0 (GraphPad Prism; San Diego, CA, USA). The unpaired student *t*-test was employed for statistical analysis comparing two groups, whereas the one-way ANOVA was utilized for comparing 2 < groups. The Shapiro–Wilk test was utilized to study the normality of the data, and the F test was used to compare the variance of data in the unpaired student *t*-tests. The *p*-values below 0.05 were statistically significant. The tumor bulk RNA data obtained from the GEO database was processed using the “*preprocessCore*” package in R software (version 4.3.2); heatmap and dendrogram clustering methods were provided to detect and exclude the potential samples not appropriately sequenced. The GeneMANIA (https://genemania.org/) was leveraged to investigate the potential interactions between the studied genes with the “*Max Resultant Genes*” equal to zero.

## Results

### Predicting the targets of hsa-miR-181a-5p

**Figure 1:**
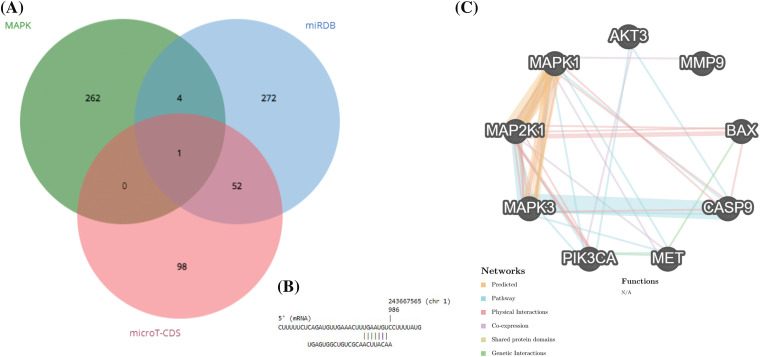
hsa-miR-181a-5p involvement in glioblastoma development. (A) Identifying the potential target of hsa-miR-181a-5p. (B) The interaction between hsa-miR-181a-5p and *AKT3*. (C) The interaction between studied genes based on the GeneMANIA database.

Based on the miRPathDB v2.0 database, Reactome, WikiPathways, and KEGG datasets, hsa-miR-181a-5p can regulate the MAPK pathway (all *p*-values < 0.01) (data are not shown). Based on these results, we attempted to identify which genes of the MAPK pathways can be targeted by hsa-miR-181a-5p; it has been found that hsa-miR-181a-5p can target *AKT3* expression (Fig. 1A,B). According to GeneMANIA, there are considerable interactions between *AKT3* with the studied genes in the present study (Fig. 1C).

### hsa-miR-181a-5p and AKT3 expression in glioblastoma tissues

**Figure 2:**
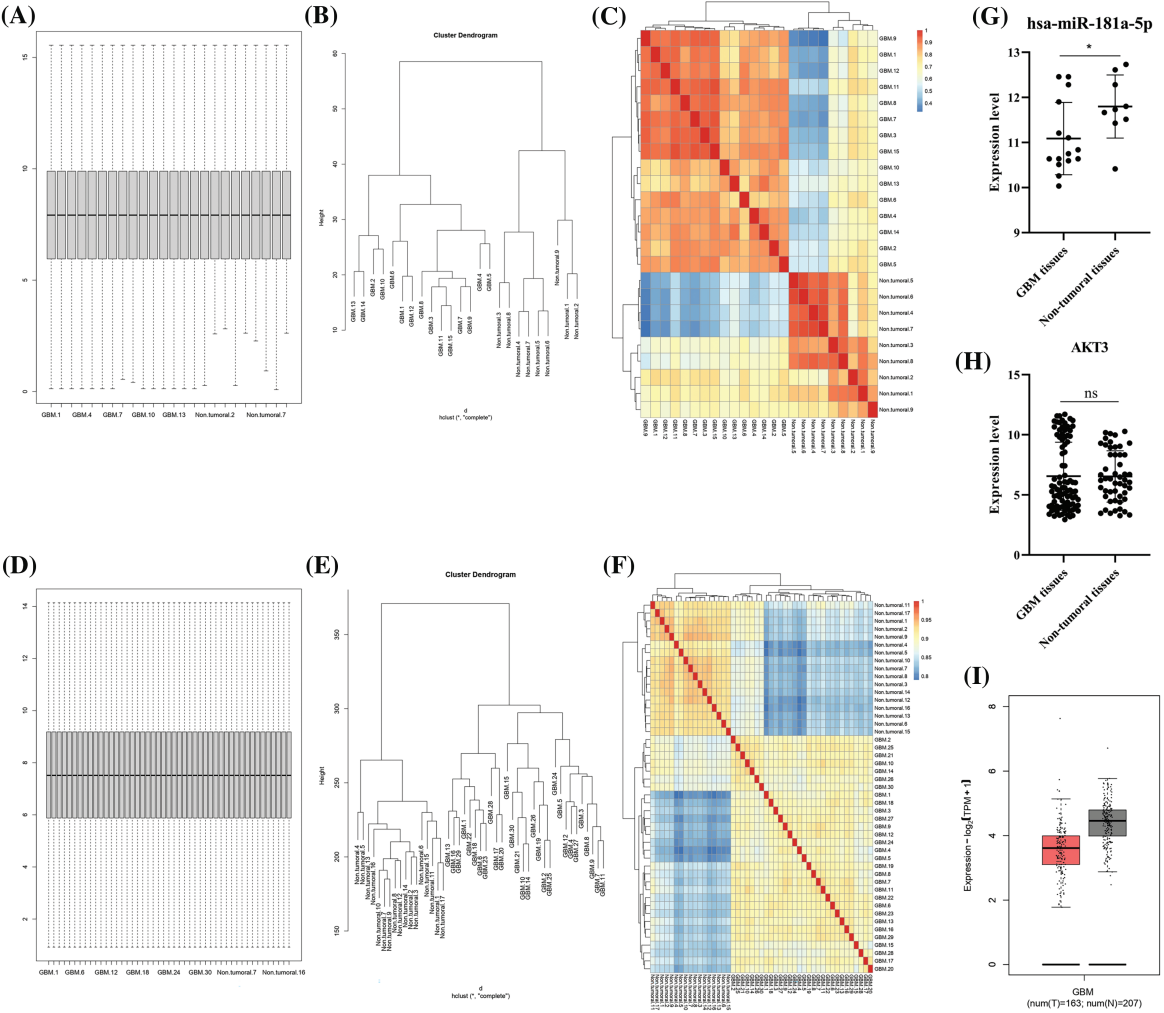
Hsa-miR-181a-5p and *AKT3* expression in glioblastoma and non-tumoral brain tissues. (A) The box plot of the GSE158284 dataset. (B) The cluster dendrogram of the GSE158284 dataset. (C) The heatmap of the GSE158284 dataset. (D) The box plot of the GSE108474 dataset. (E) The cluster dendrogram of the GSE108474 dataset. (F) The heatmap of the GSE108474 dataset. (G) hsa-miR-181a-5p expression in glioblastoma based on the GSE158284 dataset. (H) *AKT3* expression in glioblastoma based on the GSE108474 dataset. (I) *AKT3* expression in glioblastoma based on the TCGA-GTEx dataset. Ns: Not significant; *p < 0.05.

After quantile normalization of expression values, it has been found that glioblastoma and non-tumoral tissues have distinct expression patterns (Fig. 2A–F). hsa-miR-181a-5p is significantly downregulated in glioblastoma tissues compared to control (Fig. 2G); however, there is no statistically significant difference between *AKT3* expression value between glioblastoma and non-tumoral tissues (Fig. 2H–I).

### hsa-miR-181a-5p mimic effect on the cell viability, chemo-sensitivity, and stemness of U373 cells

**Figure 3:**
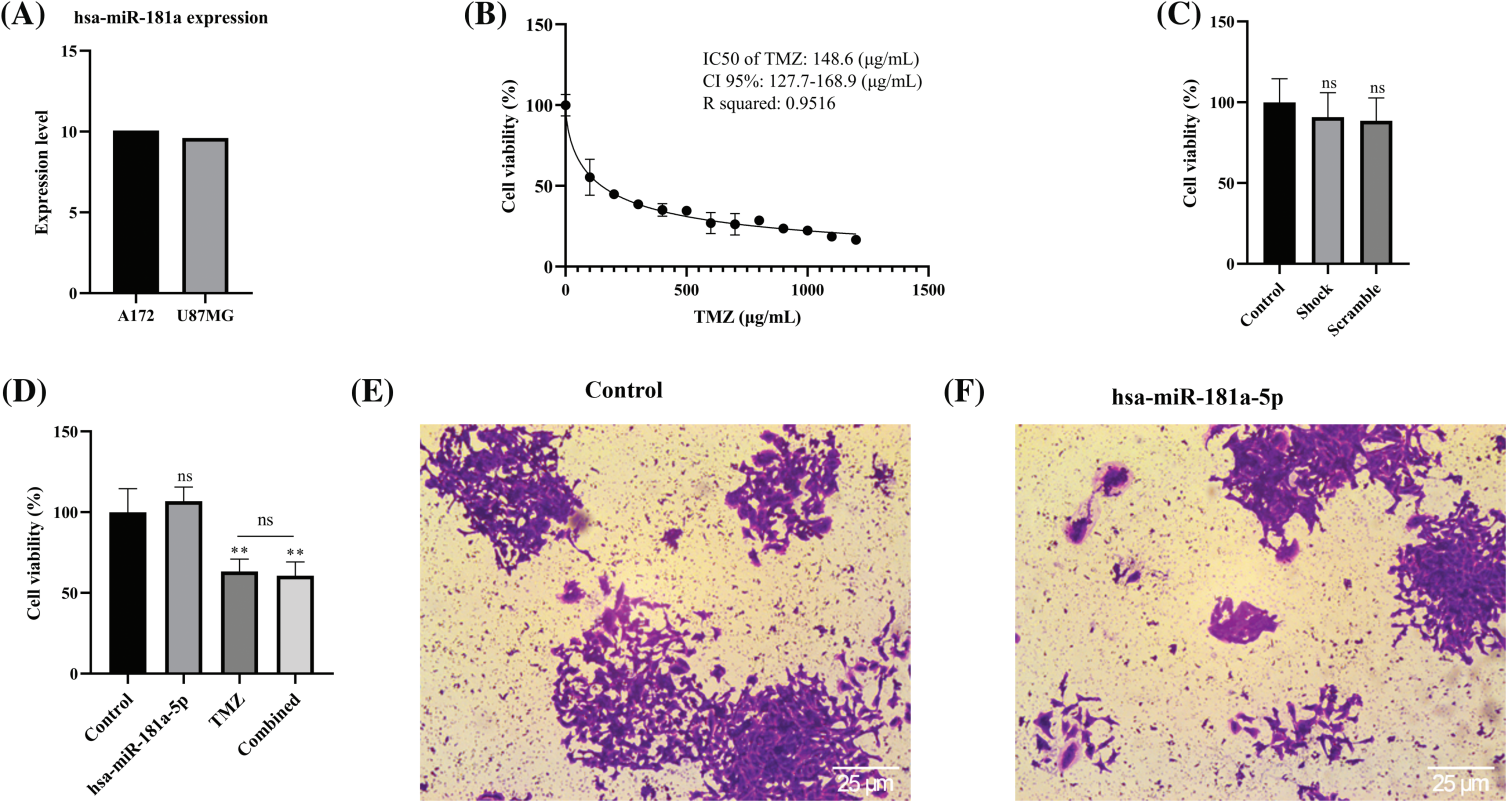
Hsa-miR-181a-5p significance in cell viability, chemo-sensitivity, and stemness of U373 cells. (A) miR-181a expression in the U87MG and A172 cells based on the CCLE dataset. (B) The IC50 of temozolomide on U373 cells. (C) The effect of the transfection process on the cell viability of U373 cells. (D) The effect of hsa-miR-181a-5p ectopic expression on the cell viability and chemosensivity of U373 cells. (E and F) The effect of hsa-miR-181a-5p mimics on the stemness of U373 cells. Ns: Not significant; **p < 0.01.

Based on the CCLE database, hsa-miR-181a-5p expression is lower in U87MG cells than in A172 cells (Fig. 3A). Based on the study by Rezaei et al., hsa-miR-181a-5p is significantly lower in U373 cells than U87MG cells [26]. Therefore, we selected the U373 cell line for subsequent assays.

With an R squared of 0.9516, our results have shown that the IC50 of temozolomide on U373 cells is 148.6 (µg/mL) (Fig. 3B). To study the potential cytotoxicity of our transfection process, we performed the MTT assay on control, shock, and scramble groups. It has been found that the transfection process does not affect the cell viability of U373 cells (Fig. 3C). In addition, hsa-miR-181a-5p does not significantly affect the viability of U373 cells, nor does it increase the chemo-sensitivity of U373 cells to temozolomide (Fig. 3D). Furthermore, hsa-miR-181a-5p does not considerably alter the clonogenicity of U373 cells (Fig. 3E,F).

### hsa-miR-181a-5p mimic decreases the migration of U373 cells

**Figure 4:**
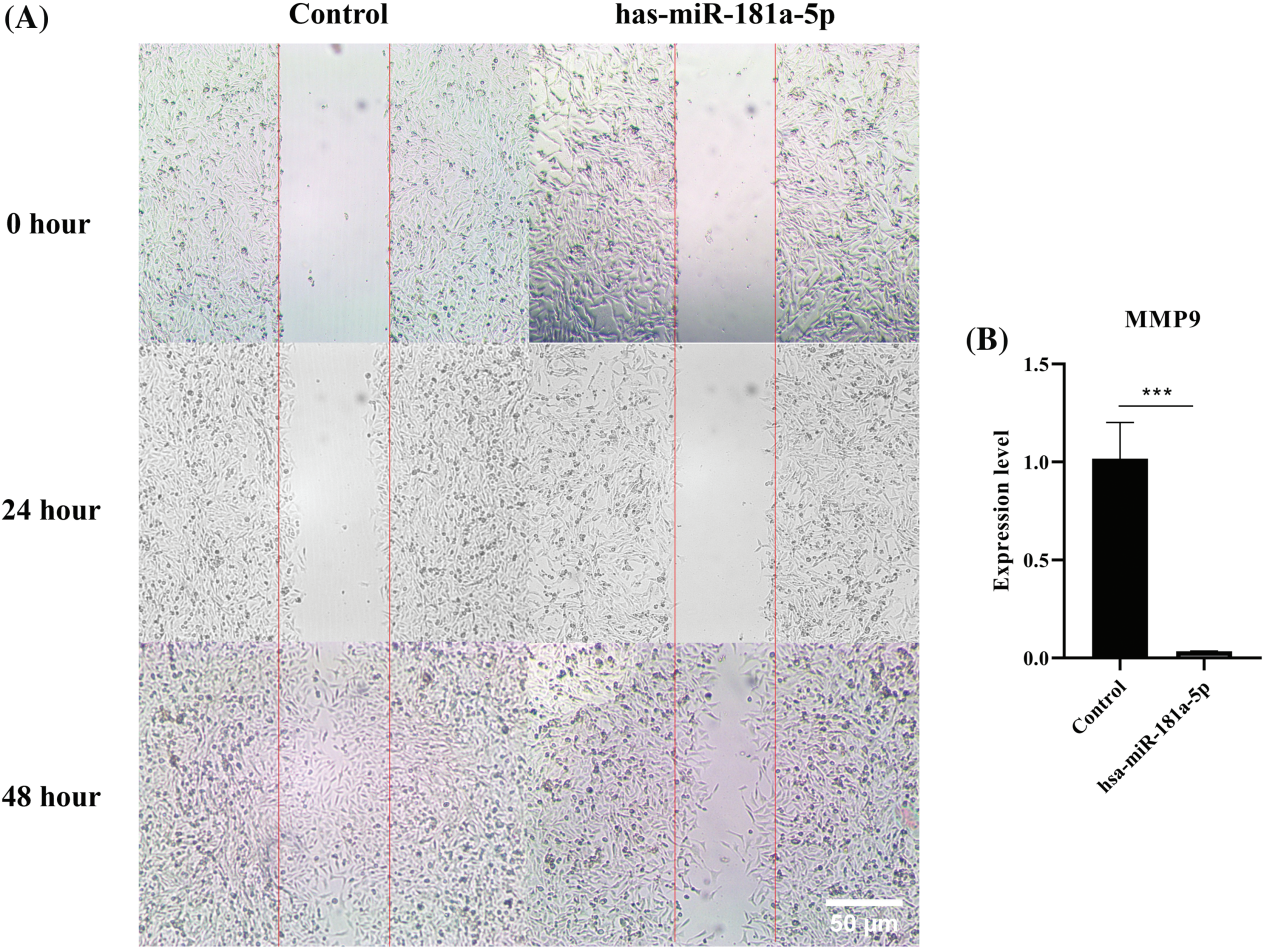
The effect of the hsa-miR-181a-5p mimics on the migration of U373 cells. (A) the migration assay, (B) *MMP9* expression in the groups. ***p < 0.001.

According to the results of the migration assay, hsa-miR-181a-5p inhibits the migration of U373 cells by a considerable margin (Fig. 4A). hsa-miR-181a-5p significantly downregulates *MMP9* expression of U373 cells as well (*p*-value = 0.0008) (Fig. 4B).

### hsa-miR-181a-5p ectopic expression increases the apoptosis rate of U373 cells

**Figure 5:**
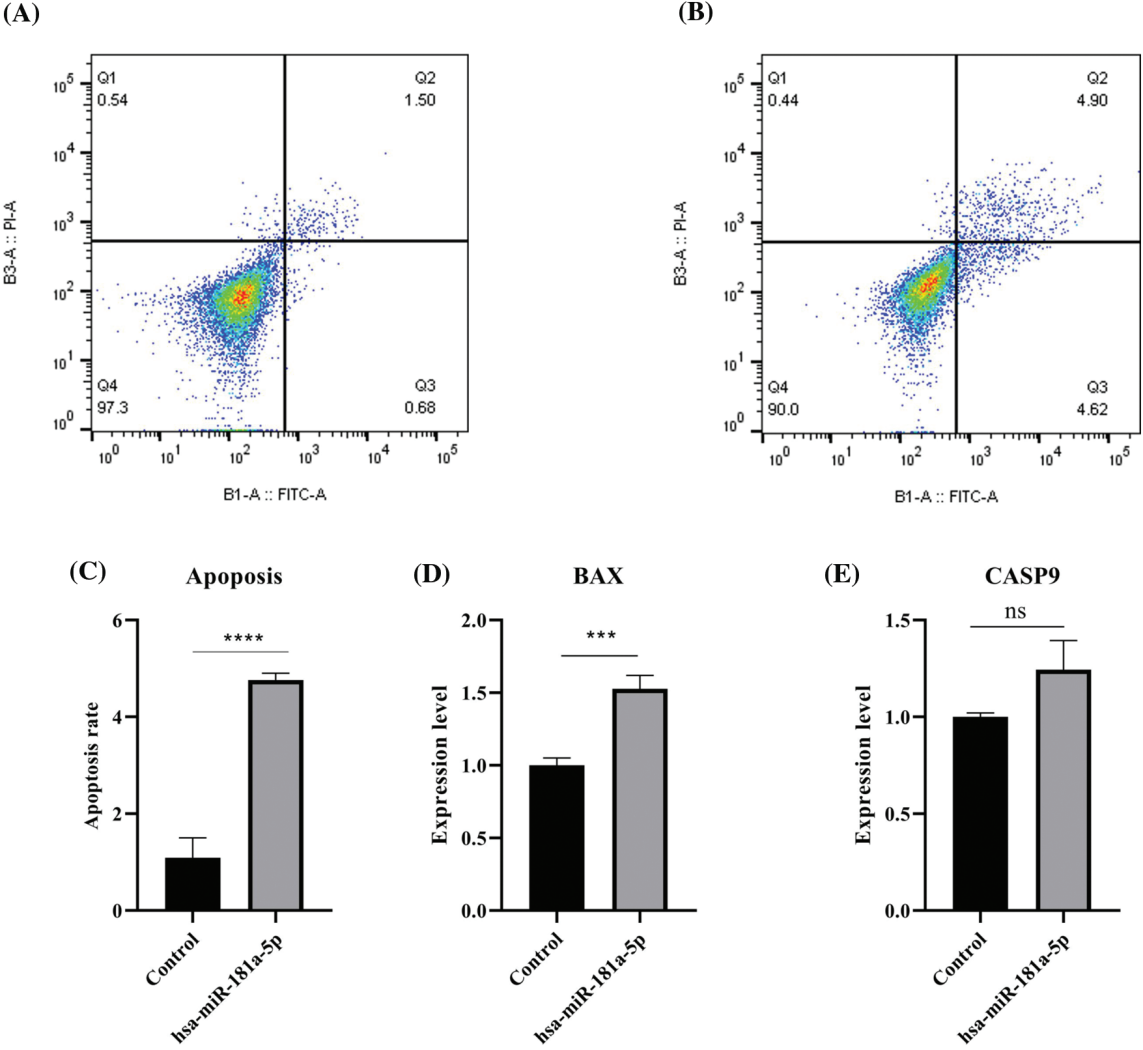
The effect of hsa-miR-181a-5p mimics on the apoptosis rate of U373 cells. (A) control group. (B) hsa-miR-181a-5p group. (C) Comparing the apoptosis rate of studied groups. (D) The mRNA expression of *BAX* in the studied groups. (E) The mRNA expression of *CASP9* in the studied groups. Ns: Not significant; ***p < 0.001; ****p < 0.0001.

It has been found that hsa-miR-181a-5p mimics increase the apoptosis rate of the malignant cells (*p*-value < 0.0001) (Fig. 5A–C). Although hsa-miR-181a-5p mimic does not significantly alter *CASP9* expression (*p*-value > 0.05), it significantly upregulates *BAX* expression in U373 cells (Fig. 5D–E).

### hsa-miR-181a-5p mimic arrests the cell cycle of U373 cells at sub-G1

**Figure 6:**
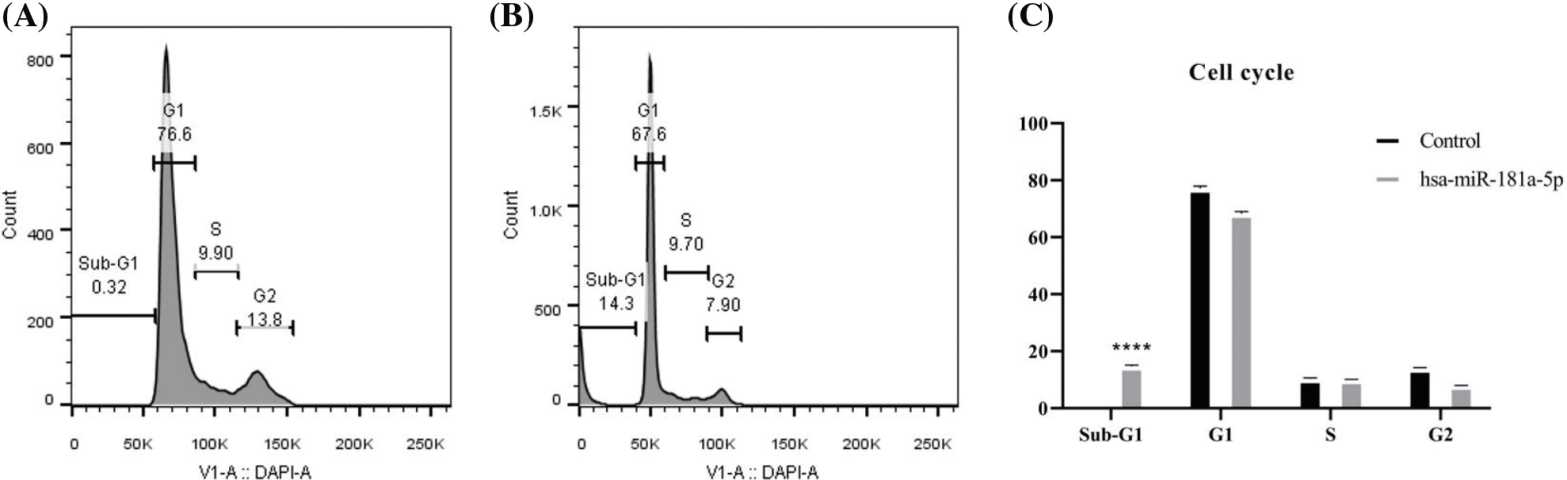
The effect of the hsa-miR-181a-5p ectopic expression on the cell cycle phase distribution of U373 cells. (A) Control group. (B) hsa-miR-181a-5p group. (C) Comparing cell cycle phases’ distribution among populations under study. ****p < 0.0001.

Afterward, we studied the effect of hsa-miR-181a-5p ectopic expression on the cell cycle of U373 cells. hsa-miR-181a-5p mimics arrest the cell cycle of U373 cells at the sub-G1 phase (*p*-value < 0.0001) (Fig. 6A–C).

### hsa-miR-181a-5p and the MAPK pathway in U373 cells

**Figure 7:**
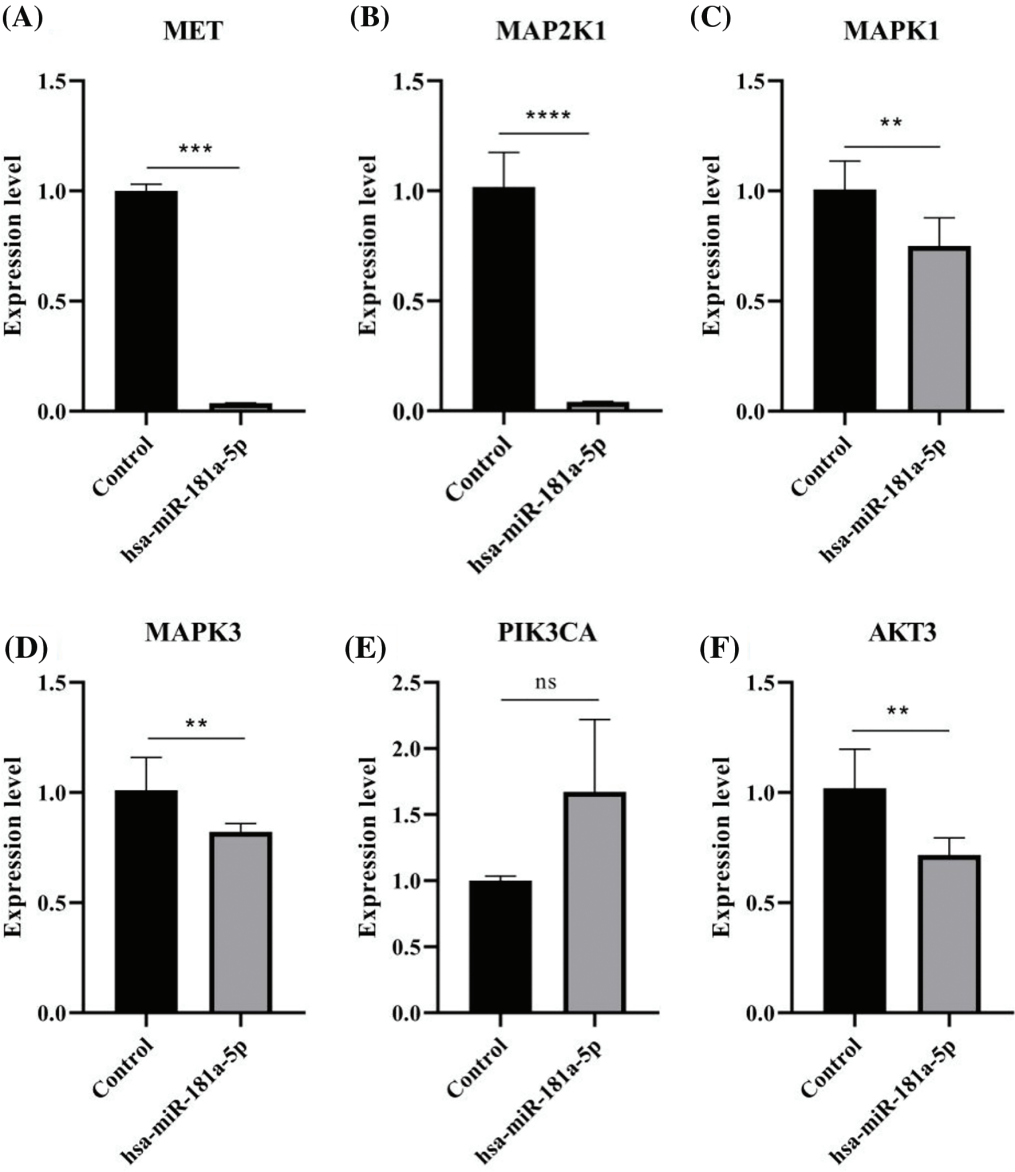
The effect of the hsa-miR-181a-5p mimics on the MAPK pathway of U373 cells. (A) The mRNA expression of *MET*. (B) The mRNA expression of *MAP2K1*. (C) The mRNA expression of *MAPK1*. (D) The mRNA expression of *MAPK3*. (E) The mRNA expression of *PIK3CA*. (F) *AKT3* mRNA expression in the groups. Ns: Not significant; **p < 0.05; ***p < 0.001; ****p < 0.0001.

In the next step, we examined the effect of hsa-miR-181a-5p ectopic expression on the MAPK and PI3KA/Akt pathways in U373 cells. hsa-miR-181a-5p ectopic expression significantly downregulates *MET*, *MAP2K1*, *MAPK1*, *MAPK3*, and *AKT3* expression in U373 cells (Fig. 7). However, hsa-miR-181a-5p does not significantly alter *PIK3CA* expression (Fig. 7).

## Discussion

Although there have been remarkable advances in understanding glioblastoma pathogenesis, the prognosis of glioblastoma patients is still undesirable. In light of the significant role of miRs in regulating various genes, tumor-suppressive miRs can be a promising targeted therapy for glioblastoma. Therefore, there is a need to identify tumor-suppressive miRs and their underlying cellular signalings and mechanisms in glioblastoma development. The present study has demonstrated that hsa-miR-181a-5p ectopic expression can regulate the MAPK pathway, increase the apoptosis rate, decrease the migration, and arrest the cell cycle of U373 cells at the sub-G1 phase. However, hsa-miR-181a-5p does not improve the chemo-sensitivity of U373 cells to temozolomide.

MiRs are non-coding, short RNA molecules with an average length of 22 nucleotides [3]. The canonical miR biogenesis is the dominant pathway for miR biogenesis. In this pathway, the transcription of DNA sequences leads to primary miRs development. Then, the DGCR8 and Drosha–mediated processing of primary miRs produces precursor miRs; finally, the RNase III endonuclease Dicer-mediated processing of primary miRs produces mature miRs. The interaction of miRs with the 3′ untranslated regions of mRNAs regulates the gene expression; however, miRs can also interact with the coding sequence and promoter of genes [27]. Dysregulated miR expression is one of the culprits for glioblastoma development [28]. Upregulated oncogenic miRs and downregulated tumor-suppressive miRs can liberate oncogene mRNA expression and decrease tumor-suppressive mRNA expression, leading to tumor growth. Since miRs can regulate a wide range of mRNA expression, they can be considered a promising targeted therapy approach [29]. Therefore, identifying the role of dysregulated miRs and regulating their expression can be a promising therapeutic strategy. This approach can be upregulating tumor-suppressive miRs and/or downregulating oncogenic miRs. In this regard, the advances in tumor-bulk RNA sequencing and microarrays can have high value for identifying dysregulated genes.

hsa-miR-181a-5p, along with miR-181b, miR-181c, and miR-181d, belongs to the miR-181 family; hsa-miR-181a-5p is located in chromosome 1 [30]. The anti-tumoral effects of hsa-miR-181a-5p have been noticed in cancers like esophageal adenocarcinoma [31], glioblastoma [26], non-small cell lung cancer [7], and cervical cancer [32]. Wen et al. have reported that the miR-181a-5p level is downregulated in relapsed glioblastoma compared to primary glioblastoma. Also, U251 cell migration and invasion are enhanced by an inhibitor of miR-181a-5p [13]. Compared to normal brain tissues and glial cell lines, miR-181a-5p is reduced in glioma tissues and cell lines; hsa-miR-181a-5p promotes apoptosis and inhibits the migration of U251 cells [11]. Moreover, Shi et al. have shown that miR-181a-5p can reduce the invasion of U87 cells and increase the apoptosis rate of U87 cells [14]. Based on our results, hsa-miR-181a-5p increases apoptosis rate, decreases migration, and arrests the cell cycle at the sub-G1 phase in U373 cells. Despite the mentioned studies, there are insufficient studies investigating the effect of hsa-miR-181a-5p on the MAPK pathway, as a well-established oncogenic signaling pathway, in glioblastoma cells.

The Ras-Raf-MEK-ERK pathway is a well-studied oncogenic pathway stimulated in glioblastoma cells [33]. Stimulating receptor tyrosine kinases, like HGFR/c-MET, activates the Ras-Raf-MEK-ERK signaling factors, which results in glioblastoma growth [34]. In this regard, the phosphorylation of tyrosines Y1349 and Y1356 on the c-MET stimulates the MAPK pathway, which results in invasion and cell cycle progression in cancerous cells [35]. Schäfer et al. have shown that hsa-miR-181a-5p ectopic expression downregulates pERK1/2 in glioblastoma cells [36]. Also, there is evidence that hsa-miR-181a-5p downregulates the expression of *AKT1*, a signaling factor of the MAPK pathway, in glioblastoma cells [26]. Following extensive *in-silico* investigations, it has been found that hsa-miR-181a-5p can regulate the MAPK pathway via suppressing *AKT3*. It has been found that hsa-miR-181a-5p can decrease *AKT3* expression in U373 cells. Besides, hsa-miR-181a-5p downregulates the expression of *MET*, *MAP2K1* (MEK1), *MAPK1* (ERK2), and *MAPK3* (ERK1), resulting in potential regulation of the MAPK pathway in U373 cells.

The current study has several strengths. First, extensive *in-silico* research was performed before the *in-vitro* study to investigate the hsa-miR-181a-5p-mediated pathway in glioblastoma. Second, a throughout literature review was performed to compare the obtained results with the current knowledge. However, there are also some limitations to this study. First, we could only study our research question on a single cell line, although we discussed the findings of other researchers who used other glioblastoma cell lines for miR-181a mimics. Second, we could not include Western blot assay to assess protein expression levels and phosphorylation states. Third, we could not perform *in-vivo* studies. Fourth, although multiple databases validated the *in-silico* results of this study, there is a likelihood of off-target effects, and we could not perform experiments like double luciferase assay to further validate the obtained results. Even though this study provides a valuable reference for future works regarding the significance of hsa-miR-181a-5p in glioblastoma development, the obtained results should be carefully interpreted with these potential biases. Further confirmatory studies in other glioblastoma cell lines are warranted. Also, validation research at the protein level and direct target interactions between miR-181a-5p and *AKT3*, a signaling factor of the MAPK pathway, via wet experiment techniques (e.g., double luciferase assay) are planned.

## Conclusion

The *in-silico* studies show that miR-181a-5p is downregulated in glioblastoma tissues and can regulate the MAPK pathway. The *in-vitro* studies demonstrate that miR-181a-5p mimics decrease the migration, increase the apoptosis rate, and arrest the cell cycle at the sub-G1 phase in U373 cells. It has been found that miR-181a-5p can suppress the MAPK pathway via downregulating *MET*, *MAP2K1*, *MAPK1*, *MAPK3*, and *AKT3* expression in U373 cells.

## Data Availability

The datasets analyzed during the current study are available in the GSE158284 (https://www.ncbi.nlm.nih.gov/geo/), GSE108474 (https://www.ncbi.nlm.nih.gov/geo/), TCGA-GTEx (http://gepia.cancer-pku.cn/), and CCLE (https://sites.broadinstitute.org/ccle/).
